# Development and validation of a nomogram prognostic model for patients with neuroendocrine tumors of the thymus

**DOI:** 10.1111/1759-7714.13556

**Published:** 2020-07-12

**Authors:** Jia‐Yu Tang, Hui‐Jiang Gao, Guo‐Dong Shi, Xiao‐Kang Guo, Wen‐Quan Yu, Hua‐Feng Wang, Yu‐Cheng Wei

**Affiliations:** ^1^ Department of Thoracic Surgery Affiliated Hospital of Qingdao University, Qingdao University Qingdao China

**Keywords:** Neuroendocrine tumors, nomogram, prognosis, thymus

## Abstract

**Background:**

The purpose of this study was to analyze the clinical characteristics and prognostic survival of patients with neuroendocrine tumors of the thymus (NETTs), and to develop and validate a nomogram model for predicting the prognosis of patients.

**Methods:**

We conducted a retrospective analysis of patients with neuroendocrine tumors of the thymus in the Surveillance, Epidemiology, and End Results (SEER) database in the United States between 1988 and 2016. Cox scale risk regression analysis, the Kaplan‐Meier method and log‐rank test were used to carry out the significance test to determine the independent prognostic factors, from which a nomogram for NETTs was established. C‐index and calibration curve were used to evaluate the prediction accuracy of the model. External validation of the nomogram was performed using data from our center.

**Results:**

A total of 254 patients with NETTs were collected in the SEER database. In the multivariable analysis, T stage, tumor grade, surgery, and chemotherapy were found to be independent factors affecting the prognosis of patients (all *P* < 0.05). A nomogram model was constructed based on these variables, and its c‐index was 0.707 (0.661–0.752). The c‐index results showed that the nomogram model had better authentication capability than the eighth edition of the tumor, node, metastasis (TNM) staging system and Masaoka‐Koga (MK) staging system. The calibration curve showed that the model could accurately predict patient prognosis.

**Conclusions:**

The study established a nomogram model that predicted the overall survival rate of one‐, three‐ and five‐years, and used the survival prediction model to optimize individualized therapy and prognostic follow‐up through risk stratification.

## Graphical abstract

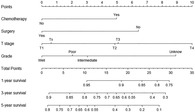

The neuroendocrine tumors of the thymus is a rare invasive tumor with a poor prognosis, and visualization models are needed to provide patients with appropriate individualized treatment strategies.In this study, we established and validated a new Nomogram model to predict the survival of patients with neuroendocrine tumors of the thymus.

## Introduction

Thymic neuroendocrine tumors originate from the neuroendocrine cells present in the whole body, and are capable of producing a variety of biogenic amines, with a strong aggression, resulting in a low overall patient survival rate.^1^ A representative retrospective analysis study to date was an analysis of 205 patients with thymic neuroendocrine tumors identified in the ITMIG and European Society of Thoracic Surgeons (ESTS) databases. This study showed that tumor excision and pathological staging are the main prognostic factors;^2^ however, survival rates varied widely in patients at the same stage. Some scholars speculate that there are other factors that may improve the accuracy of individual survival predictions on this basis, such as age, gender, histology classification and treatment‐related factors.^3^, ^4^ In the present study, we attempted to establish a nomogram model for thymic neuroendocrine tumors using the Surveillance, Epidemiology, and End Results (SEER) database, combined with known clinicopathologic variables, and to provide advice on the establishment of the tumor, node, metastasis (TNM) staging for the ninth edition of the thymus. In addition, we used a separate queue in the domestic database for external validation.

## Methods

### Study participants

**Figure 1:**
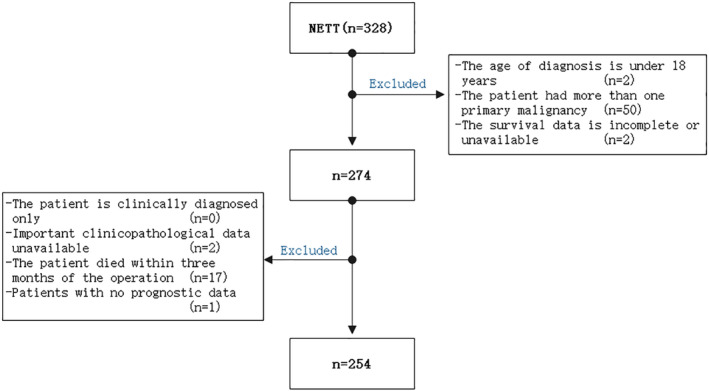
Study the flow chart of the queue screening process.

A retrospective analysis was performed on 254 eligible patients from the SEER database between 1988 and 2016. Time of survival was defined as from the date of diagnosis to the last follow‐up (31 December 2019) or the date of death. Patients were selected according to the corresponding histological codes (8150‐8157, 8240‐8246, 8249) and primary site (C37.9, C38.1) in the International Classification of Diseases codes, third edition (ICD‐O‐3). Clinical variables included gender, age, race, histological subtype, size, marriage, AJCC T stage (extension of tumor), surgical status, chemoradiotherapy, and survival‐related information. Patients was excluded from the study if: (i) they were under 18 years of age at diagnosis; (ii) the patient had more than one primary malignant tumor; (iii) survival data were incomplete or unavailable; (iv) patients were only clinically diagnosed; (v) lack of important clinical pathology information; (vi) the patient died within three months of the operation (to eliminate perioperative mortality); and (vii) patients had no prognostic data. All patients were from the SEER database and the Affiliated Hospital of Qingdao University database (Fig 1).

According to the latest edition of the World Health Organization thymic tumor classification in 2015, thymic neuroendocrine tumors are no longer classified as well differentiated and poorly differentiated. In fact, they are classified as low‐grade (typical carcinoids), intermediate (atypical carcinoids) or high‐grade cancers (small cell carcinoma and large cell neuroendocrine carcinoma), so the data of 254 cases of large cell neuroendocrine tumor and small cell carcinoma were divided into the poor‐differentiation group, the atypical carcinoid group into the intermediate‐differentiation group, and the typical carcinoid were classified as the well‐differentiation group.^5^

### Statistical analysis

Excel 2016 was used to preliminarily organize data, In the training set, use Kaplan‐Meier to estimate the survival curve of different variable values and compare them using log‐rank tests. Multivariable Cox regression analysis was performed using SPSS Version 23.0 (Armonk, NY: IBM Corp), the variable of the significant *P* < 0.05 enters the multivariate analysis to determine the independent prognostic factors that predict total survival. The rms package in R software 3.6.1 (http://www.r-project.org/) were used to construct a visual model based on independent prognostic variables of the training dataset, and the accuracy of the nomogram model was measured according to the Harrell's c‐index. A self‐service method of 1000 resamples was set up to calculate the correction curve by regression analysis.^6^ Finally, calibration diagrams were used to correct the difference between nomogram prediction and observational survival, indicating the accuracy of the nomogram for one, three and five years of OS. A calibration chart along the 45‐degree line means a perfect model that suggests a great deal of consistency between the predicted and actual results. Comparisons between the nomogram and other staging systems were evaluated by the c‐index, which were performed with the rcorrp.cens package in Hmisc in R.

## Results

Of the 328 patients with thymic neuroendocrine tumors from 1988 to 2016, 254 (77.4%) who met the inclusion criteria were enrolled in the training set (Table 1). In addition, 25 NETTs patients from the center were included in the validation set between 2014 and 2019. Among eligible patients, 83 (32.7%) were female; median age was 55 years (18–88 years); 123 (48.4%) and 104 (40.9%) were those receiving radiation and chemotherapy, respectively. Well‐graded and moderately differentiated tissues (66.1%) were the most common tumor grades after median follow‐up times of 107 months (range: 4–292 months) and 35 months (range: 4–66 months) for the training and validation cohorts, respectively. The median OS was 74 months, and the one‐,three‐ and five‐year OS rates were 82.7%, 67.4% and 52.8% in the training cohorts, respectively. In the study, most patients underwent surgery. It is important to note that the SEER database does not contain variables used to determine the order of surgery and chemotherapy, or to elucidate the drugs used in chemotherapy.

**Table 1:** Characteristics of neuroendocrine tumors of the thymus patients in each cohort

	Training cohort		Validation cohort	
Characteristics	N	%	N	%
Size (cm)				
<7	92	36.2	15	60
≥7	102	40.2	10	40
Unknown	60	23.6	0	0
Age				
<40	47	18.5	3	12
40–49	41	16.1	6	24
50–59	61	24.0	6	24
60–69	55	21.7	8	32
≥70	50	19.7	2	8
Gender				
Male	171	67.3	19	76
Female	83	32.7	6	24
Race				
White	189	74.4	0	0
Black	20	7.9	0	0
Others	45	17.7	25	100
Status				
Single	81	31.9	0	0
Married	165	65.0	25	100
Unknown	8	3.1	0	0
Radiotherapy				
No	131	51.6	19	76
Yes	123	48.4	6	24
Chemotherapy				
No	150	59.0	17	68
Yes	104	41.0	8	32
Grade				
Well	110	43.3	3	12
Intermediate	58	22.9	14	56
Poor	46	18.1	8	32
Unknown	40	15.7	0	0
T stage				
T1	72	28.3	10	40
T2	28	11.0	6	24
T3	75	29.5	7	28
T4	59	23.2	1	4
Tx	20	8.0	1	4
Surgery				
No	71	27.9	2	8
	183	72.1	23	92
Survival status				
Alive	108	42.52	18	72
Dead	146	57.48	7	28

**Figure 2:**
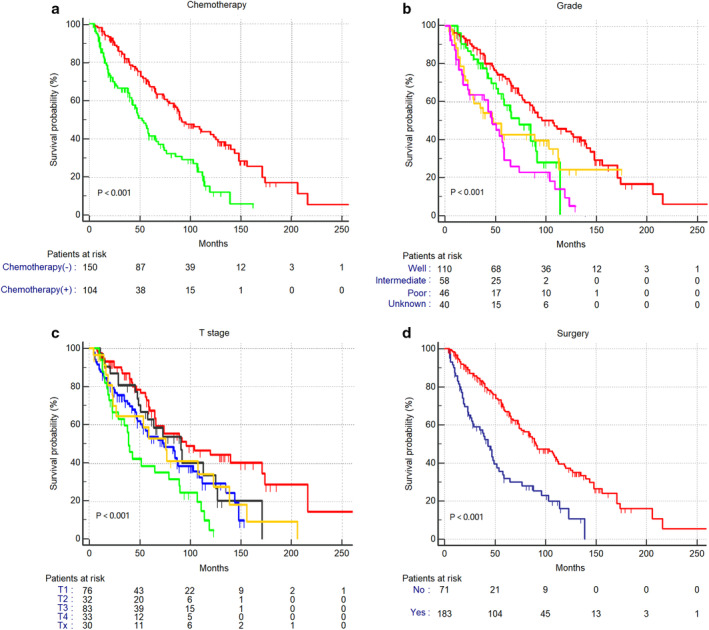
Survival analysis of patients with thymic neuroendocrine tumor by ***Chemotherapy***, Chemotherapy (−)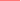, Chemotherapy (+)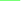 (**a**); ***Grade***, 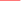
Well, 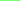
Intermediate, 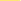 Poor, 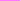 Unknown, (**b**); ***T Stage***, 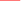
T1, 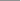
T2, 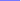
T3, 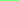, T4
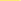
Tx (**c**); ***Surgery***, 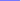 No, 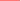 Yes (**d**).

Univariable analysis showed gender (*P* = 0.037), size (*P* = 0.002), histological subtype (*P* < 0.001), chemotherapy (*P* < 0.001), T staging (*P* < 0.001) and surgery (*P* < 0.001) was an important predictor of survival (Table 2). The relevant clinicopathological factors were adjusted for multivariable analysis. By statistical calculation, it was found that only histological subtype (*P* = 0.008), chemotherapy (*P* = 0.039), T stage (*P* = 0.0024) and surgery (*P* = 0.013) were independent factors affecting the prognosis of patients (Table 2). Notably, patients' age and tumor size were not independent risk factors for prognosis. Low grade (well‐differentiated) tumors, early‐stage tumors, nonchemotherapy, or complete resection were variables that significantly improved prognostic survival (Fig 2).

**Table 2:** Univariable and multivariable analysis of overall survival in the training cohort

	Univariable			Multivariable		
	HR	95% CI	*P*‐value	HR	95% CI	*P*‐value
Size (cm)			0.002			0.520
<7	Reference					
≥7	1.595	1.070–2.376	0.022	1.282	0.837–1.965	0.190
Unknown	2.156	1.408–3.302	0.000	1.160	0.682–1.972	0.402
Age			0.878			
<40 years	Reference					
40–49 years	1.207	0.692–2.108	0.507			
50–59 years	1.096	0.641–1.873	0.737			
60–69 years	1.27	0.755–2.135	0.367			
≥70 years	1.272	0.755–2.144	0.366			
Gender			0.037			0.138
Male	Reference					
Female	1.447	1.023–2.046	0.037	1.316	0.916–1.891	0.138
Race			0.291			
White	Reference					
Black	0.889	0.578–1.367	0.593			
Others	0.526	0.235–1.178	0.118			
Status			0.961			
Single	1.13	0.443–2.878	0.798			
Married	1.138	0.461–2.811	0.779			
Unknown	Reference					
Radiotherapy			0.278			
No	Reference					
Yes	1.203	0.862–1.681	0.278			
Chemotherapy			0.000			0.039
No	Reference					
Yes	2.149	1.532–3.015	0.000	1.52	1.021–2.264	0.039
Grade			0.000			0.008
Well	Reference					
Intermediate	1.572	0.971–2.543	0.065	1.276	0.773–2.109	0.341
Poor	1.839	1.159–2.917	0.010	1.208	0.723–2.017	0.471
Unknown	2.892	1.844–4.537	0.080	2.25	1.410–3.591	0.001
T stage			0.001			0.024
T1	Reference					
T2	1.599	0.906–2.824	0.105	1.473	0.781–2.780	0.231
T3	1.899	1.212–2.976	0.005	1.463	0.867–2.468	0.154
T4	3.088	1.834–5.198	0.000	2.187	1.305–3.664	0.003
Tx	1.855	1.022–3.367	0.042	1.002	0.453–2.216	0.997
Surgery			0.000			0.013
No	Reference					
Yes	0.407	0.286–0.579	0.000	0.577	0.373–0.893	0.013

Indicates *P* < 0.05. CI, confidence interval; HR, hazard ratio.

**Figure 3:**
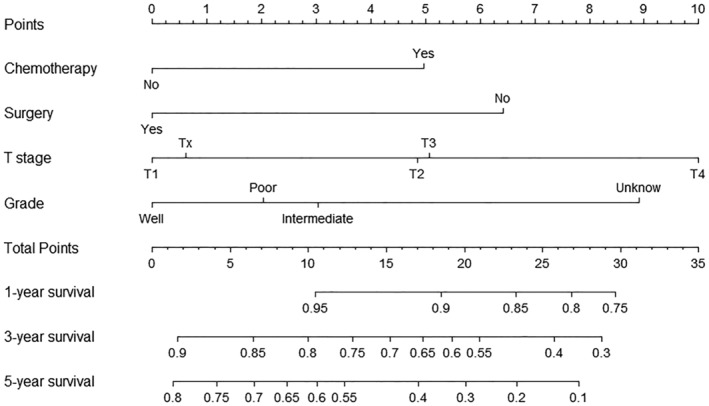
Visual nomogram prediction model.

**Figure 4:**
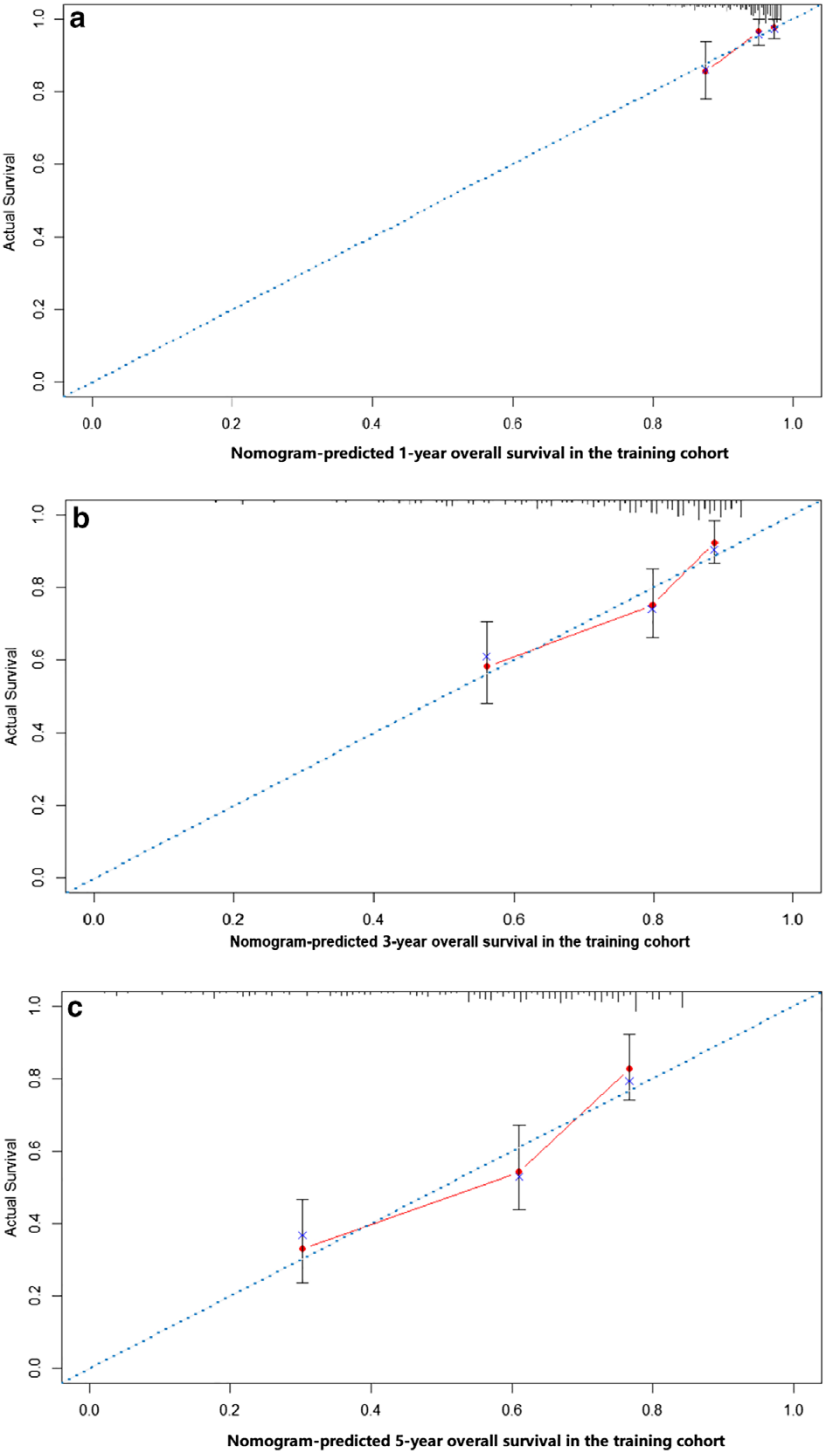
The calibration curves of the nomogram‐predicted (**a**) one‐year, (**b**) three‐year and (**c**) five‐year overall survival in the training cohort.

**Figure 5:**
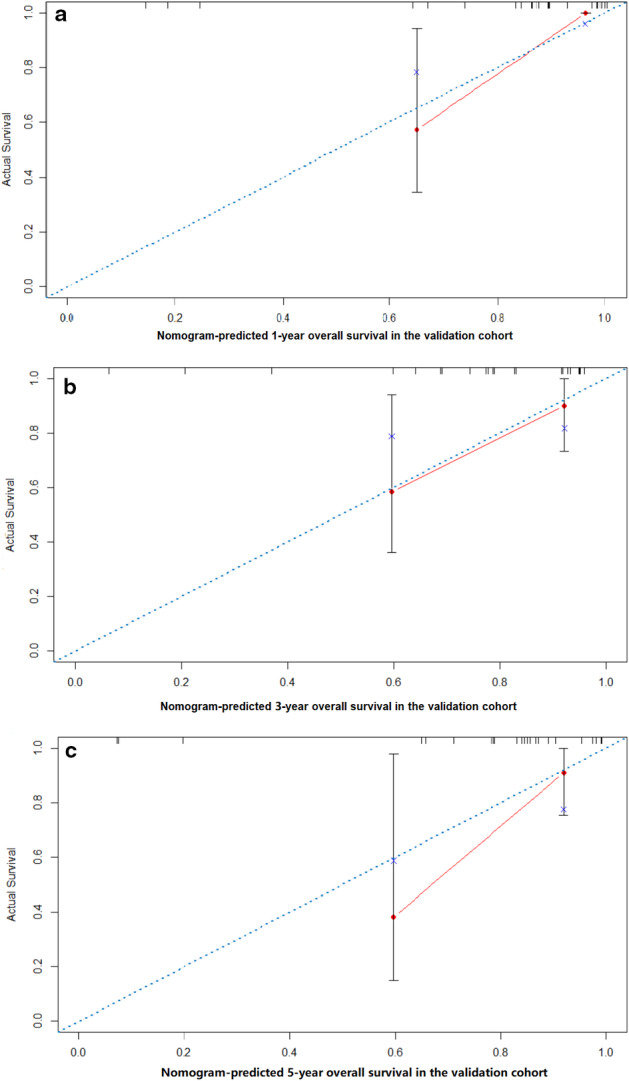
The calibration curves of the nomogram‐predicted (**a**) one‐year, (**b**) three‐year and (**c**) five‐year overall survival in the validation cohort.

A nomogram model was constructed based on the prognostic factors selected in the training dataset to predict one‐, three‐ and five‐year survival rates for thymic neuroendocrine tumors (Fig 3). The prediction model showed that T stage had the greatest effect on prognosis, followed by grade, surgery, and chemotherapy. Each level of each variable was assigned a grade score. According to the scores corresponding to different values of each variable, the total scores of individuals were calculated accordingly, and the one‐year, three‐year and five‐year survival rates of individuals obtained. The calibration diagram verified by bootstrap resampling showed a good consistency between the predicted survival and actual survival (Fig 4). In the bootstrap resampling cohort, the c‐index was 0.707 (95% CI: 0.660–0.753) in the training cohort and 0.791(95% CI: 0.646–0.937) in the validation cohort, indicating that the nomogram model was a good prediction model. The calibration chart for the external validation cohort also showed a good consistency between the actual overall survival rate and the overall survival rate predicted by the nomogram model (Fig 5).

## Discussion

Since thymic neuroendocrine tumors differ significantly in the survival of individual patients, the Masaoka‐Koga (MK) staging system or the TNM staging system cannot be accurately used to predict survival.^7^, ^8^, ^9^ Although some prognostic models have been studied,^4^, ^10^ nomogram models for thymic neuroendocrine tumors have not been developed. Therefore, in our study, we attempted to develop a nomogram model to predict and validate long‐term survival rates for individualized treatments.

The data for the training set came from the public SEER database in the United States, which consists of a total of 18 patient records based on population and geographically distinct cancer registries that cover 28% of the U.S. population. This database is one of the world's recognized authoritative sources of follow‐up data for cancer patients and represents Standards and Advanced Technology or Healthcare in the United States.^11^ Large sample sizes and extensive geographic distribution of patients ensure the representativeness and universality of the training set. We confirmed that histology, T staging, and whether surgery and chemotherapy were independent prognostic factors with univariable analysis and subsequent multivariable analysis. The findings in our study were highly consistent with previous reports on risk factors for thymic neuroendocrine tumors.^2^, ^7^, ^12^ It is worth noting that N stage is also an important factor in many cancers, ^13^, ^14^ and some earlier studies have also suggested that lymph node examination and dissection could have an effect on the patient's prognosis process.^15^, ^16^ However, due to the limitations of this study, there are no further study on N stages, but this will be the focus of our future research.

The nomogram model is a graphical representation of a complex statistical prediction model, which integrates multiple predictors to express the relationship between variables in the prediction model.^14^, ^17^ Therefore, the nomogram model has been described as an important part of modern medical decision‐making, and even applied in clinical practice as a new standard.^14^, ^18^, ^19^, ^20^ In fact, there is increasing evidence that the nomogram model plays a more important role in predicting cancer prognosis than the classic TNM staging system. According to Figs 4 and 5, our model showed good consistency on the prediction training set, and the nomogram on the validation set had a satisfactory consistency with the actual observations in the prediction of the annual survival rate. Harrell's c‐index for the established nomogram to predict OS 0.706 (95% CI: 0.660–0.753), was obviously higher than that of AJCC eighth edition staging system 0.696 (95% CI: 0.647–0.745; *P* < 0.01) and MK staging system 0.697 (95% CI: 0.648–0.746; *P* < 0.01). Therefore, we found that compared with the TNM staging system and some previous prognostic models, the constructed nomogram model represented a more accurate prognostic model.

Similar to other SEER‐based studies, this study had some limitations. First, the SEER database mainly collects the characteristics of the Western countries, and the versatility of the model needs to be further verified. Second, compared to large‐scale and prospective randomized trials, the recommended rating of retrospective studies is suboptimal. Third, important clinical pathology parameters are not available in the SEER dataset, such as surgical margin status, lymphatic osmosis, vascular immersion, nerve infiltration and some important molecular factors (MEN1 gene mutation, chromogranin and neuron‐specific enolase),^21^, ^22^ which will have an impact on the reliability of prognostic analysis.

In conclusion, a neuroendocrine tumor is a rare invasive tumor with a poor prognosis. According to our study, the significant effects it may have on the tumor include T staging, tumor histological classification, whether surgical resection is possible, and whether assisted chemotherapy should be performed. Instead, we have built and validated a new nomogram model to predict the survival of patients with neuroendocrine tumors of the thymus. In clinical work, doctors could use this model to more accurately assess the long‐term survival status of patients and provide appropriate individualized treatment strategies for specific patients.

## Disclosure

The authors declare that there are no conflicts of interest.
